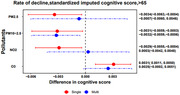# Association of Air Pollution with Memory Decline Later in Life in the United States

**DOI:** 10.1002/alz.089016

**Published:** 2025-01-09

**Authors:** Boya Zhang, Jennifer Weuve, Kenneth M Langa, Jennifer D'Souza, Adam Szpiro, Jessica D Faul, Carlos Mendes de Leon, Jiaqi Gao, Joel D. Kaufman, Lianne Sheppard, Jinkook Lee, Richard A. Hirth, Sara Adar

**Affiliations:** ^1^ University of Michigan, Ann Arbor, MI USA; ^2^ Harvard T.H. Chan School of Public Health, Boston, MA USA; ^3^ Boston University School of Public Health, Boston, MA USA; ^4^ University of Washington, Seattle, WA USA; ^5^ Georgetown University, Washington DC, DC USA; ^6^ University of Southern California, Los Angeles, CA USA

## Abstract

**Background:**

Emerging evidence suggests that air pollution exposure might diminish the cognitive health of older adults. Although many studies have reported that air pollution is associated with increased dementia risk, associations with the process of cognitive decline have been more heterogeneous.

**Method:**

We used biennial data between 2000 to 2016 from respondents>65 years in the Health and Retirement Study (HRS), a national, population‐based cohort in the United States, to study associations of air pollution with cognitive decline. We assessed episodic memory as a composite score of performance on immediate and delayed recall tests combined with proxy‐reported memory function. We standardized the memory score at baseline and during follow‐up based on its baseline mean (0.66) and standard deviation (0.69). We used spatiotemporal models to predict 10‐year average particulate matter (PM_2.5_, PM_10‐2.5_), nitrogen dioxide (NO_2_) and ozone (O_3_) at participant residences before each interview. Associations with rates of memory decline were estimated by generalized estimating equation regression, adjusting for individual demographics, area‐level characteristics, time, and spatial trends.

**Result:**

Among the 19,063 participants during an average of 7.4±5.3 years of follow‐up time, the mean age at baseline was 71.0±7.3 years. The mean (interquartile range, IQR) PM_2.5,_ PM_10‐2.5_, NO_2_ and O_3_ concentrations were 12.2 (3.6) µg/m^3^, 9.4 (4.6) µg/m^3^, 11.2 (7.8) ppb and 26.6 (3.7) ppb respectively. In single‐pollution models, we observed consistently steeper decline in episodic memory scores with higher PM_2.5_, PM_10‐2.5_ and NO_2_ long‐term concentrations (all approximately ‐0.003 standard units/year per IQR, 95%CI: ‐0.006, ‐0.0004), whereas the reverse was observed with higher exposures to O_3_ (0.003 standard units/year per IQR, 95%CI: 0.001, 0.005)_._ Associations were changed to null for PM_2.5_ and NO_2_, and slightly attenuated for O_3_ after adjustment for other pollutants in the multi‐pollutant model.

**Conclusion:**

This study suggests that long‐term exposure to air pollution might be associated with cognitive decline among older adults though the results differed by pollutant.